# Biomechanical Challenges of Olecranon Fractures in Patients With Parkinson’s Disease: A Case Analysis of Neuro-Orthopedic Collaborative Intervention Strategies

**DOI:** 10.7759/cureus.98493

**Published:** 2025-12-04

**Authors:** Qijia Liu, Jiarui Wan, Yang Li, Lei Li, Hao Wang, Yongjun Hu, Shiyang Liao, Keqing Xu

**Affiliations:** 1 Clinical Medicine, Bengbu Medical University Graduate School, Bengbu, CHN; 2 Orthopedics, The First Affiliated Hospital of Anhui University of Science and Technology, Huainan, CHN; 3 Clinical Medicine, The First Affiliated Hospital of Anhui University of Science and Technology, Huainan, CHN; 4 Orthopedics, The First Affiliated Hospital of University of Science and Technology of China, Hefei, CHN; 5 Orthopedics, Bengbu Medical University Graduate School, Bengbu, CHN; 6 Orthopedics, Hebei Medical University, Shijiazhuang, CHN

**Keywords:** deep brain stimulation, neuro-orthopedic collaborative strategy, olecranon fracture, parkinson's disease, quantitative assessment

## Abstract

In the intricate landscape of Parkinson's disease (PD), patients face a notably elevated risk of fixation failure due to the motor dysfunction characteristic of this condition. This narrative presents a compelling case of recurrent olecranon fractures in a patient with PD. It highlights the clinical implications of a neuro-orthopedic collaborative strategy that is meticulously anchored in quantitative assessment. A 71-year-old male with PD, Unified Parkinson's Disease Rating Scale III (UPDRS III) score 45 and modified Ashworth scale grade 2, sustained a comminuted fracture of the right olecranon (classified as Mayo Clinic type IIB) following a fall. The initial fixation, performed with titanium cables, was followed by a secondary wire fixation. Both fixations ultimately failed, compromised by the biomechanical stresses caused by the tremor and muscle rigidity intrinsic to PD. The third intervention, which combined a plate with tension band wiring, resulted in successful fixation. After undergoing deep-brain stimulation (DBS), which alleviated his tremor symptoms, the patient sustained a contralateral olecranon fracture during rehabilitation. This fracture was effectively managed using the same fixation protocol. A neuro-orthopedic collaborative strategy was carefully implemented, using the UPDRS-III and modified Ashworth scale scores to quantify neurological impairment and guide the choice of fixation methods. Ultimately, successful fixation was achieved after three procedures on the right elbow and one on the left. Notably, post-DBS, the patient showed marked improvement in tremor symptoms and achieved a near-normal range of motion (flexion: 130°, extension lag: 10°) and an excellent Mayo Elbow Performance Score (MEPS) of 95/100 in both elbows during follow-up assessments. This neuro-orthopedic collaborative strategy, firmly based on quantitative assessment, effectively addresses the complex biomechanical challenges inherent in fracture management for patients with PD. This approach not only improves fixation success but also significantly enhances functional outcomes, reflecting meticulous planning and execution at every step.

## Introduction

Parkinson's disease (PD) is a prevalent degenerative disorder of the nervous system, predominantly characterized by resting tremor, rigidity, and bradykinesia, with its pathological basis rooted in the degeneration of dopaminergic neurons within the substantia nigra [1]. As the condition advances, individuals with PD frequently face an increased risk of falls, with incidence rates soaring to an alarming 60% to 80%. Furthermore, the occurrence of fractures among these patients is observed to be 2.3 times greater than that of the general population [2]. Moreover, the resting tremor and lead-pipe rigidity, characteristics of PD, generate persistent, abnormal biomechanical stresses across a fracture site. These forces can lead to cyclic loading of the hardware, resulting in screw loosening within osteoporotic bone, metal fatigue, and ultimately fixation failure. This sequence presents a unique and significant challenge in orthopedic trauma management for this population.

Olecranon fractures of the ulna, constituting 8% to 11% of elbow fractures, are often caused by direct trauma or falls. The therapeutic approach typically emphasizes anatomical reduction and stable fixation [3]. Patients with PD often exhibit pronounced motor dysfunction, manifesting as tremors, muscle rigidity, and freezing of gait episodes. These symptoms increase the risk of inadvertent falls and subsequent bone fractures [4]. Furthermore, the abnormal muscle tone and rigidity in patients with PD make diagnosing olecranon fractures particularly challenging, complicating clinical evaluations. These factors contribute to a markedly elevated rate of internal fixation failure. However, the precise underlying mechanisms remain unclear [4,5]. In cases of recurrent internal fixation failure, clinical management becomes increasingly complex. However, the existing literature reveals a scarcity of reports addressing such cases, underscoring the imperative for further scholarly inquiry.

The distinctiveness of the current case lies in its thorough examination of the intricate biomechanical mechanisms linking PD with upper limb fractures, accompanied by a meticulously crafted stepwise intervention strategy. This study introduces a neural-orthopaedic collaborative intervention approach, illuminating novel pathways for the tailored treatment of such multifaceted cases. Through detailed biomechanical analysis, the research underscores the critical significance of early neural regulation and the biomechanical benefits inherent in combined fixation. It serves as a foundational reference for clinical treatment and paves the way for future investigations. Nevertheless, the broader applicability of this methodology requires validation through larger sample sizes.

## Case presentation

We present a case of a 71-year-old male with a six-year history of PD managed with a regimen of selegiline 5 mg twice daily, pramipexole 0.25 mg three times daily, amantadine 100 mg twice daily, and Madopar 200 mg twice daily. This dosage was maintained peri-operatively in close consultation with the neurology team to minimize motor fluctuations that could jeopardize fixation stability. His clinical status is indicated by a Unified Parkinson's Disease Rating Scale (UPDRS-III) [6] score of 45 and a Modified Ashworth scale [7] grade of 2 while on this medication regimen. The patient’s main complaints included the progressive tremors in his limbs, persistent muscle rigidity, and episodes of gait freezing. His clinical condition involves moderate to severe motor dysfunction, which necessitates the use of a walking stick for outdoor activities. He had originally planned to undergo scheduled deep-brain stimulation (DBS) surgery in the neurosurgery department. However, while walking, he fell and suffered a fracture of the right elbow. This injury led to his transfer to the orthopedic department. Both initial and subsequent falls were characterized as forward-pulling falls due to postural instability and gait freezing, a common mechanism in PD leading to direct impact on the elbow. 

**Figure 1:**
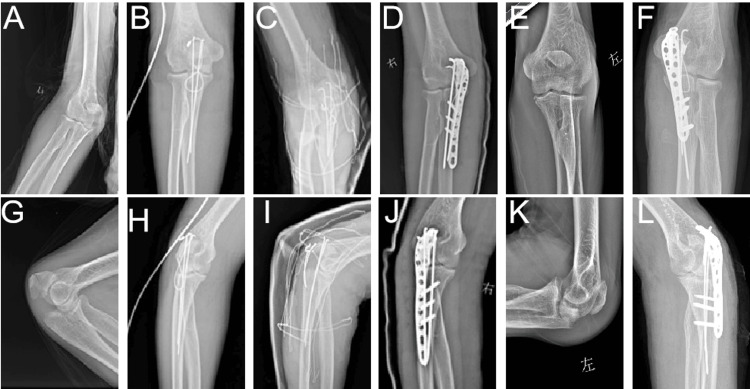
X-ray Images of Both Elbows Prior to and Following Surgical Intervention (A, G) Preoperative fractures of the right elbow depicted in anteroposterior and lateral perspectives; (B, H) Initial fixation failure of the right elbow shown in anteroposterior and lateral perspectives; (C, I) Subsequent fixation failure of the right elbow presented in anteroposterior and lateral perspectives; (D, J) Recovery observed after combined fixation of the right elbow illustrated in anteroposterior and lateral perspectives; (E, K) Preoperative fractures of the left elbow represented in anteroposterior and lateral perspectives; (F, L) Combined fixation of the left elbow.

Radiographic imaging revealed a right ulnar olecranon comminuted fracture, classified as Mayo IIB (Figure 1A, 1G) [8]. Nearly nine months post-surgery, the patient experienced a second fall, resulting in a left ulnar olecranon comminuted fracture, classified as Mayo IIA (Figure 1E, 1K) [8]. The treatment process was as follows: during the initial surgical intervention for the right elbow, the ulnar olecranon fracture was treated with tension-band fixation. Subsequent radiographic evaluations conducted the day after the procedure revealed displacement of the fracture (Figure 1B, 1H). The second revision of the right elbow necessitated reassessment, during which the tension band was replaced, and external fixation combined with plaster immobilization was employed. This fixation failed three days post-operation, once again revealing fracture displacement (Figure 1C, 1I). During the third fixation attempt for the right elbow, a more robust approach was adopted, incorporating a combination of tension band fixation and plate fixation (Figure 1D, 1J). After this third fixation, the patient was transferred to the Department of Neurosurgery for deep-brain stimulation surgery, which yielded a significant improvement in the patient's tremor. Regarding the left elbow, the initial surgical intervention addressed the ulnar olecranon fracture classified as Mayo IIA. This was treated with a combination of tension band fixation and plate fixation, resulting in a stable postoperative condition (Figure 1F, 1L).

**Figure 2:**
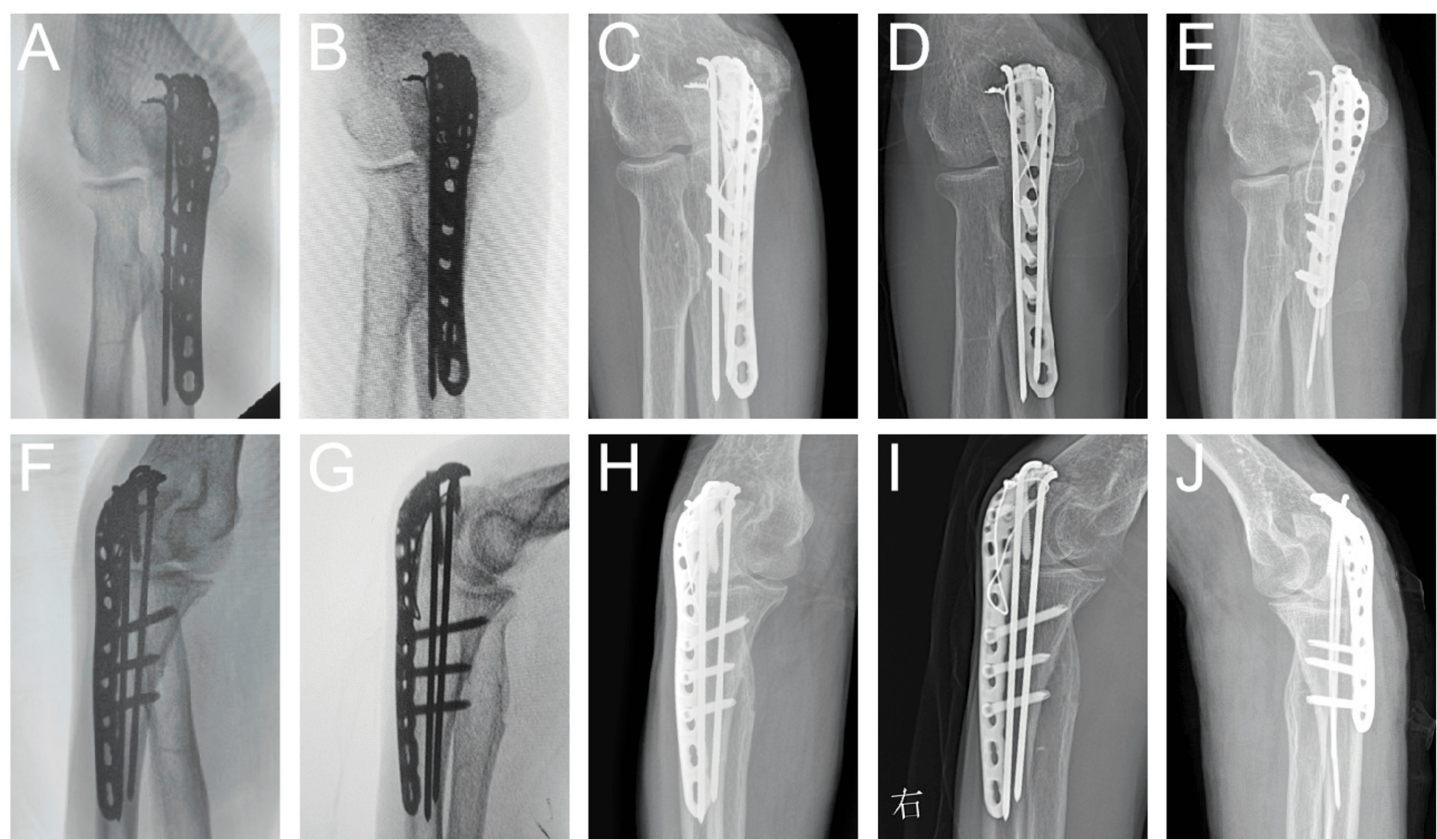
Series of follow-up radiographs. (A, F) One month postoperatively, anteroposterior and lateral projections of the right elbow; (B, G)Three months postoperatively, anteroposterior and lateral projections of the right elbow; (C, H)Five months postoperatively, anteroposterior and lateral projections of the right elbow; (D, I)Eight months postoperatively, anteroposterior and lateral projections of the right elbow; (E, J) Nearly three months postoperatively, anteroposterior and lateral projections of the left elbow.

Figure 2 shows serial postoperative radiographs demonstrating progressive fracture healing of both elbows at various follow-up intervals.

**Figure 3:**
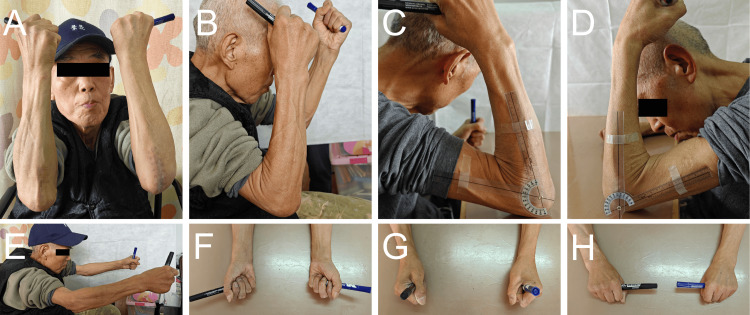
Rehabilitation status (A) left and right elbow flexion, front view; (B, C, D) left and right elbow flexion, side view; (E) left and right elbow extension, side view; (F) left and right elbow neutral position; (G) left and right elbow pronation; (H) left and right elbow supination.

Figure 3 presents clinical photographs demonstrating excellent postoperative range of motion, including flexion, extension, pronation, and supination of both elbows. Functional evaluations confirmed that both elbows could actively flex to 115°(Figures 3A-3D). Rotational function was fully preserved. However, the extension was limited by approximately 10° (Figure 3E). Despite this limitation, the overall functional outcome fully met the needs of daily life (Figures 3F-3H). 

Based on the UPDRS-III [6,9] and the modified Ashworth scale [7,10], we propose a neuro-orthopedic collaborative intervention management strategy (Table 1). The choice of treatment plans no longer depends solely on the type of fracture but must also consider and address the biomechanical challenges posed by PD-related neurological dysfunction. First, the severity of these challenges should be quantified through scoring, followed by the selection of treatment plans that effectively address them. This neuro-orthopedic collaborative intervention is an advanced and precise medical concept centered on the use of objective quantitative assessment tools, such as the UPDRS-III and modified Ashworth scale, to guide and optimize a comprehensive intervention strategy that integrates therapeutic methods from both the neurology and orthopedic departments. The ultimate goal is to maximize improvements in motor function and quality of life for patients.

**Table 1:** Neuro-orthopedic collaborative intervention management strategy. UPDRS-III: Unified Parkinson's Disease Rating Scale III, DBS: deep brain stimulation, PD: Parkinson's disease.

Degree	Rating criteria	Core risks	Intervention plan
Mild	UPDRS-III ≤ 32 points, modified Ashworth ≤ 1+ level	Low risk of fixation failure, good rehabilitation compliance	Optimize PD medication treatment + muscle tone band fixation + early protective rehabilitation training
Moderate	UPDRS-III 33–58 points, modified Ashworth 2–3 level	Significant tremor interference, high muscle tone, high risk of fixation failure	DBS + medication adjustment + combined fixation using a muscle tone band and steel plate + delayed and cautious rehabilitation (external fixation with plaster)
Severe	UPDRS-III ≥ 59 points, modified Ashworth ≥ 4 level	High risk of fixation failure, unable to cooperate with rehabilitation, poor healing environment	Multi-modal neuromodulation + olecranon resection or total elbow joint replacement + emphasis on pain management and complication prevention

## Discussion

This case report presents the treatment process of a 71-year-old male with PD who had multiple fractures of the ulnar epicondyle. It systematically elaborates on the crucial role of a multidisciplinary neurosurgical intervention strategy in addressing the biomechanical challenges associated with PD. The following discussion focuses on treatment evaluation and functional outcomes. It integrates quantitative assessment and elements of multidisciplinary collaboration, aiming to provide evidence-based guidance and clinical insights for managing similar complex cases.

Treatment challenges and biomechanical factors of PD-related fractures 

Patients with PD have a significantly higher risk of falls and fracture incidence due to motor disorders, such as tremors, muscle rigidity, and postural instability, compared to the general population [11]. In this case, the UPDRS-III score of 45 points (moderate to severe motor disorders) and the modified Ashworth grade of 2 (significant muscle spasticity) exacerbated the risk of failure of orthopedic internal fixation. These challenges, including fixation failure due to tremors and muscle rigidity, were vividly demonstrated in the treatment process for the right elbow fractures. The first surgery (a titanium cable figure-of-eight internal fixation) failed because it did not adequately consider the cyclic load generated by the persistent PD tremors (Figure 1B, 1G). Studies by Chou et al [12] and Zhan et al [13] also confirmed that the failure rate of internal fixation in PD patients is higher than in non-PD patients due to osteoporosis caused by neuromuscular dysfunction and the additional biomechanical stress introduced. The second revision technique (a wire figure-of-eight fixation combined with plaster external fixation with the elbow flexed at 90°) aimed to balance fixation and function, but still failed due to increased joint stress caused by muscle rigidity and micro-movements induced by tremors (Figure 1C, 1I). This failure may be attributed to two factors. First, the rigidity of PD muscles increased the stress around the joint, interfering with the stability of the fixation device. Second, although the flexed elbow position is beneficial for functional recovery by maintaining joint mobility and reducing stiffness, it cannot effectively counteract the micro-movements and dislocation forces caused by tremors [12-14]. Notably, the patient's initial neurological scores, UPDRS-III (45), and modified Ashworth scale (2), indicated a moderate biomechanical risk profile, per our proposed strategy (Table 1). However, the first two interventions employed techniques suited for a mild risk profile. This disconnect between the patient's neurological status and the chosen fixation method directly contributed to the failures.

Based on the lessons learned from the previous two failures, a combined figure-8 fixation using steel plates and wires was adopted in the third attempt. This was accompanied by a plaster cast maintaining the elbow in a semi-flexed position (Figure 1D, 1J). Through biomechanical optimization, such as adjusting fixation angles and tension to enhance stability, secure fixation was ultimately achieved. After DBS surgery, tremors improved, leading to reduced involuntary movements and thereby decreasing the mechanical load on the fixation device. Based on the success in the third attempt, this fixation technique was then applied to the left elbow fractures (Figure 1E, 1F, 1K, 1L) using the same dual fixation strategy. The postoperative functional recovery was excellent, with the range of motion of both elbows restored to near-normal levels (flexion: 115°, extension lag: 10°). In the most recent follow-up, the Mayo Elbow Performance Score (MEPS)[15] was 95 points, indicating an excellent functional outcome.

Precise intervention strategies guided by quantitative assessments 

The innovation of this study lies in integrating objective quantitative tools, UPDRS-III, and modified Ashworth scales, into the treatment decision-making framework. Through these assessment tools, the severity of neurological dysfunction is quantified. This provides specific guidance for selecting rigid fixation based on quantified neurological and biomechanical indicators. Specifically, high UPDRS-III scores (33-58 points) and high Ashworth scores (2-3) indicate the need to adopt a combined rigid fixation method, such as plate and wire fixation, to resist biomechanical stress. As a result, this strategy was directly successful when applied to the left elbow, effectively avoiding repeated fixation failures on the right elbow by better addressing biomechanical demands (Figure 2E, 2J). ZhuParris A et al [9] developed the MDS-UPDRS scale, and Meseguer-Henarejos AB et al. [10] validated the Ashworth scales, providing reliable evidence that demonstrates the application value of precision medicine" in complex patients.

The value and functional outcomes of comprehensive management through neurosurgical and orthopedic collaboration

This case highlights the crucial role of multidisciplinary collaboration (MDT) in optimizing outcomes. Neurosurgery uses DBS surgery to alleviate core motor symptoms (such as tremors), thereby creating a favorable environment for orthopedic fixation. Orthopedics directly addresses structural issues by biomechanically optimizing fixation techniques, such as dual fixation, which uses two methods to enhance stability. This collaborative model breaks down the barriers between specialized fields and aligns closely with the recently advocated comprehensive medical concept [16,17]. The success of this collaborative model was ultimately verified through systematic follow-up. The imaging examination showed that the fractures of the ulnar epiphysis of both elbows had clinically healed (Figure 2). Functional evaluations confirmed that both elbows could actively flex to 115°(Figures 3A-3D). Rotational function was fully preserved. However, extension was limited by approximately 10°(Figure 3E). Despite this limitation, the overall functional outcome fully met the needs of daily life (Figures 3F-3H). This outcome strongly demonstrates that this collaborative strategy ultimately achieved the core goal of maximizing improvement in the patient's motor function and quality of life.

Clinical implications and limitations

It is important to acknowledge the full spectrum of management options for olecranon fractures. Non-operative management, such as functional bracing, is a recognized and appropriate strategy for elderly patients with low physical activity demands who have minimally displaced fractures, as highlighted in a recent systematic review [18]. In addition, novel suture fixation techniques have emerged as a less invasive alternative for osteoporotic bone, potentially reducing the risk of hardware-related complications [19]. However, for this specific patient, non-operative management was deemed unsuitable due to the comminuted and displaced nature of both fractures (Mayo IIA/IIB), which necessitates anatomical reduction and stable fixation to achieve functional outcomes. While suture fixation is promising, the extreme and persistent biomechanical stresses caused by this patient's severe PD tremor and rigidity (UPDRS-III: 45, Ashworth scale: 2) mandated the absolute stability provided by a combined plate and tension-band construct. Biomechanical studies have shown this construct to offer superior strength to withstand high loads [20]. Therefore, our treatment choice was a deliberate, evidence-based decision tailored to this unique biomechanical challenge.

The neuro-surgical collaborative intervention strategy proposed in this case provides a new paradigm for the management of pedicle fractures. It emphasizes that treatment decisions should integrate fracture classification, quantitative assessment of neurological function, and biomechanical risk analysis. However, as a case report, this study has certain limitations. A single sample restricts the generalizability of the conclusions. Additionally, the follow-up period is relatively short (eight months for the right elbow and three months for the left) and therefore insufficient to evaluate long-term outcomes. Although we have provided detailed pharmacological management, the optimal timing of performing DBS surgery after an acute fracture remains an area for further investigation. Nevertheless, a multi-center cohort study is needed to further validate this strategy and explore its clinical utility in patients with various neurological diseases.

## Conclusions

Based on this case analysis, we believe that the unique biomechanical characteristics and treatment needs of PD patients must be considered in fracture treatment. The integration of quantitative neurological assessments, including the UPDRS-III and the Modified Ashworth Scale, into the orthopedic decision-making process is crucial for achieving successful outcomes. Although this study provides new insights into the complexity of fracture management in PD patients, it has certain limitations, such as a small sample size and short follow-up duration. Therefore, future research needs to conduct multi-center, large-scale clinical trials to obtain more comprehensive conclusions. Future research should also focus on evaluating the long-term effects of the combined therapeutic approach of DBS and internal fixation, and exploring the applicability of this strategy in other types of fractures and in patients at different stages of PD. Furthermore, additional clinical trials will help validate the effectiveness of the neuro-orthopedic collaborative intervention strategy approach, integrating neurological and orthopedic treatments to optimize fracture management in PD patients, thereby providing a solid scientific basis for individualized treatment plans.
